# Recent trends in robot-assisted therapy environments to improve real-life functional performance after stroke

**DOI:** 10.1186/1743-0003-3-29

**Published:** 2006-12-18

**Authors:** Michelle J Johnson

**Affiliations:** 1 Medical College of Wisconsin, Dept. of Physical Medicine & Rehabilitation, 9200 W. Wisconsin Ave, Milwaukee, WI 53226, USA; 2 Marquette University, Dept. of Biomedical Engineering, Olin Engineering Center, Milwaukee, WI USA; 3 Clement J. Zablocki VA, Dept. of Physical Medicine & Rehabilitation, Milwaukee, WI, USA; 4 The Rehabilitation Robotics Research and Design Lab, Clement J. Zablocki VA, 5000 National Ave, Milwaukee, WI, USA

## Abstract

Upper and lower limb robotic tools for neuro-rehabilitation are effective in reducing motor impairment but they are limited in their ability to improve real world function. There is a need to improve functional outcomes after robot-assisted therapy. Improvements in the effectiveness of these environments may be achieved by incorporating into their design and control strategies important elements key to inducing motor learning and cerebral plasticity such as mass-practice, feedback, task-engagement, and complex problem solving.

This special issue presents nine articles. Novel strategies covered in this issue encourage more natural movements through the use of virtual reality and real objects and faster motor learning through the use of error feedback to guide acquisition of natural movements that are salient to real activities. In addition, several articles describe novel systems and techniques that use of custom and commercial games combined with new low-cost robot systems and a humanoid robot to embody the " supervisory presence" of the therapy as possible solutions to exercise compliance in under-supervised environments such as the home.

## Background

Stroke is the leading cost of disability in the USA and rehabilitation is estimated to cost $60 billion annually for the 5.4 million living with disability. Neurological impairment after stroke frequently leads to hemiparesis or partial paralysis of one side of the body. This hemiparesis can profoundly impair functional performance of activities of daily living (ADLs) such as walking, running, and eating [1]. For example, at 6 months post-stroke 50% of survivors at least 65 years old had some hemiparesis, 30% were unable to walk, and 26% were dependent in activities of daily living (ADLs).

Increasingly, robot-assisted therapy devices are used in stroke rehabilitation. Robotic tools provide opportunities to study functional adaptation after a stroke and can provide objective measurements of the time-course of changes in motor control of the affected limbs. Robot-assisted therapy permits semi-autonomous practice of therapeutic tasks [2-14].

Early examples of upper limb robots such as the MIT-MANUS therapy robots [5] were designed to permit stroke survivors to practice two-dimensional (2-D) point-to-point movements. Other examples such as the Gentle/s [6] and MIME [7] therapy robots permit stroke survivors to practice three-dimensional (3D) point-to-point reaching movements occurring in a haptic virtual environment or in the real world. Typically, to practice these movements, the stroke survivor's impaired arm is supported against gravity while he/she is asked to use the impaired hand to hold the handle of the robot and move it or permit the impaired arm to be moved through reaching exercises. The length of interventions varies, but typically consists of exposure to the robot for three to five sessions per week for 4 to 8 weeks.

Early examples of robotic lower limb robots are the GT I servo-controlled gait trainer developed and used for training in the 1990s in Germany [8,9] and the Lokomat manufactured by Hocoma AG (Switzerland) [10,11]. Typically, these systems simulate the phases of gait and modify key gait parameters such as stride length and walking speed. Often these systems are used in the rehabilitation of non-ambulatory patients such as those with SCI and partially ambulatory patients such as those with stroke and as such they often support some percentage of a patient's body-weight. Training often consists of repetitive stepping on a treadmill training three to five days per week for 4 to 8 weeks.

Preliminary studies using these upper and lower limb robotic tools demonstrate their effectiveness and their limitations. The extent of motor impairment reduction seen after upper limb robot-assisted therapy environments has been shown to be dependent on lesion size and location, and the treatment has been shown to be target-area specific, e.g., training tasks emphasizing the shoulder will improve the shoulder but not the hand. In general, these upper arm systems have mixed impact on upper limb real-life function. They can reduce motor impairment after stroke, but still have mixed impact on functioning in real life use of the upper arm [2-4]. New upper arm robotic devices including exoskeletons are being proposed to examine new training strategies that focus on using more functional training environments along with virtual environments to improve carryover and reduce gravity discoordination [12-14]. More so than in the upper limb, studies show that lower limb robot-assisted therapy environments have had more success with fewer challenges to their overall effectiveness. Results do indicate that the repetitive step training, which is by nature very task-specific and relevant to real walking, does improve reduce motor impairment and functional limitations in some patients [9,11,15]. Although not all patients benefit and there are concerns about EMG activation patterns being different from those observed during natural walking, the training seems to improve gait parameters such as gait speed and endurance.

The mixed results from robot therapy environments, especially upper limb ones, suggest that there is still a need to optimize these treatment strategies and prove that rehabilitation robot systems are worth pursuing. If we believe this is true and that these systems have the potential to decrease long-term healthcare costs for patient, then we must clarify how best to design and use them. For answers rehabilitation engineers have begun to examine the neuroscience literature on cerebral plasticity to gain some insight into the next generation of robot therapy environments. The following briefly describes some of the relevant findings from neuro-rehabilitation and neuroscience and introduce nine articles that present new robots and new control models and feedback techniques to enrich robot-assisted therapy environments.

### Cerebral Plasticity

The underlying neurological mechanisms and central nervous system recovery patterns after stroke therapy is poorly understood and this is true whether the intervention is mediated with robots or other strategies such as the Bobath method of Neuro-Development Therapy (NDT) [16]. Preliminary evidence suggests that simply moving or passively exercising the impaired limb will not lead to maximum recovery. Functional cortical reorganization and carryover of motor gains after stroke seem to be linked to therapies that involve the intense use of the impaired limb and involve the acquisition of new motor skills [19-23]. Evidence also suggests that in addition to mass-practice and use of the arm, enriched environments [17-19], highly functional and task-oriented practice environment [20-24], and highly motivating environment that increase task engagement [25-27] are important for motor re-learning and recovery after stroke. Literature supports the fact that the mechanism in mediating functional recovery seen after stroke is more than likely due to the sprouting of new synapses, the unmasking of redundant motor networks, and the re-organization of the areas around the lesion site [19].

Specifically, functional imaging studies indicate that motor recovery is characterize by the following: 1) an increase in the size of the motor and sensory areas in the lesioned hemisphere that is dedicated to the impaired limb; 2) enhance activity and recruitment in preexisting motor networks in unaffected regions and those surrounding the lesion site and in the cerebellum, and 3) a reduction the amount of activity in primary and secondary motor regions over time, especially in areas in the hemisphere ipsilateral to the lesion [24,28-32]. Similar findings have emerged from animal models of neurological plasticity [33].

**Figure 1:**
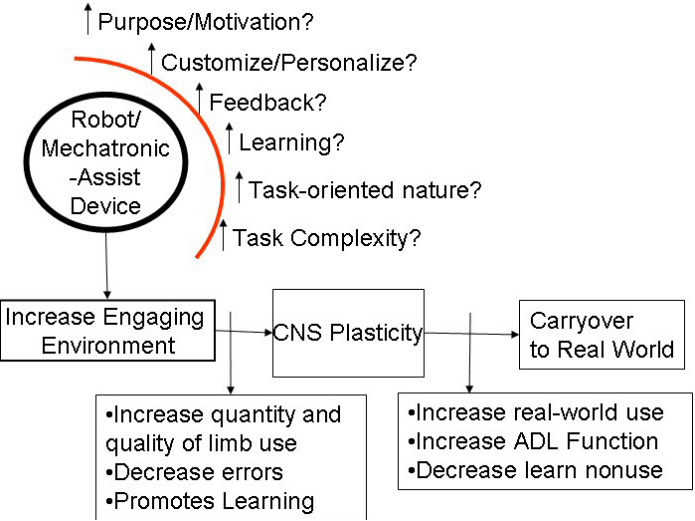
**New Ideas for Improving Robot-Assisted Therapy**. In improving robot-assisted therapy to improve carryover after stroke new methods have sought to modify the environment through enhanced feedback, personalization and task relevance.

Researchers have begun to respond to the neurological evidence and have begun to create robot-assisted therapy environments that can better capitalize on these findings and improve the likelihood of use-dependent cortical reorganization and carryover to ADL function. In this special issue, we highlight several attempts to improve the effectiveness of robot therapy environments using several extrinsic motivational techniques including feedback. Figure 1 describes the impact desired for new robotic/mechatronic assistive systems for stroke rehabilitation and some of the methods being employed. The robot-assisted environment may be modified to better engage the stroke survivor (e.g., provide extrinsic motivators), to improve its relevance to the person and the activities they do in real life (i.e., increase task-oriented nature, purpose and patient-centered), to improve feedback strategies (i.e., increase feedback of errors and results) and to improve learning strategies (i.e., employ new control strategies).

#### Enhanced Feedback in Lower Limb Gait Rehabilitation

The first set of two articles deals with lower limb robotics and demonstrate the use of biofeedback, virtual reality, and haptics to create more engaging gait training environments. The environments also provide opportunity for more complex and more functional gait training.

The article by Lunenburger and colleagues [34] discuss the use of biofeedback of the patient's gait performance to improve robot-assisted gait training. They demonstrate a novel strategy that uses sensors embedded in the robot environment to define and display the biofeedback values to the patient and therapists. In contrast, Schmidt and colleagues [35] focuses on the HapticWalker environment and uses virtual reality to create real-life walking environments. Their novel programming of the foot plates enable them to simulate versatile gait patterns such as walking up and down stairs.

#### Game-Based and Social-Based Robot-Assisted Training Trends

The next set of four articles discuss new developments in upper limb robot-assisted stroke therapy from the point of view of using game- and social-oriented activities to define motivating training environments. The articles present strategies that seek to understand and improve the use of the impaired arm in daily activities in environments away from clinical supervision. In the past, robotic and computer-assisted systems such as JavaTherapy [36] and Driver's SEAT [37], designed for clinical and home rehabilitations, have used entertainment to sustain motivation and task interest in therapy. There is still a need for home-based rehabilitation ideas that will work and deal with the challenge of cost, boredom, and compliance with prescribed exercise routines that are diverse, complex, and functional. These papers offer several novel ways to promote task-engagement and complex problem solving, two elements that are thought to be key to plasticity.

Johnson, Feng, and colleagues [38] discuss a novel Robot/computer-assisted suite of assisted devices for home-based therapy that attempts to tap into patient's need for personal and fun therapy to sustain motivation in under-supervised environments. The proposed system stresses a low-cost approach that is much needed in this field. They describe the use of distinct off-the-shelf and custom force-feedback joystick and wheel systems that are all usable with a custom-made software called Unitherapy. Also using games as a platform for training, the next article by Colombo, Pisano, and colleagues [39] demonstrate the effectiveness of two low-cost robotic systems, the planar 2-DOF robot called MULOS and a wrist robot. The combined system focused on the shoulder and elbow and wrist pronation and supination. Along with standard and custom clinical measures, they used an intrinsic motivation scale by McAuley [40] to assess the attention and interest of their stroke subjects. Their study provides further indication of the utility of low-cost, game-based platforms and new metrics that can quantify engagement.

In the article by Mataric, Eriksson, and colleagues [41] we gain a novel perspective on how non-contact robotic systems can be of use in rehabilitation of the stroke survivor. Coining the term "socially assistive robots," they demonstrate the novel use of an autonomous mobile platform programmed with several levels of feedback and monitoring capability. They demonstrate the effectiveness of the system in monitoring limb use while providing encouragement and reminders throughout a therapy session. This study provides a humanoid-like solution to the under-supervised clinical environment with the provision of the feedback via a robot embodying human qualities.

Finally in this series, Amirabdollahian, Loureiro, and colleagues [42] discuss results from using the Gentle/s robot therapy system, which is a virtual reality and haptic enhanced training environment. They examine the results using a novel multivariate regression analysis tools. Their results support the potential of better evaluation methods capable of detecting performance changes due to robot-assisted therapy systems.

#### New control and modeling strategies for Robot-Assisted Training

The next set of three articles describe solutions and ideas for improving the modeling and control of robot-assisted therapy systems to aide them in adapting patients' movements to natural and functional activities such as walking, drinking, and pinching. In the past other researchers have examined the use of error to improve motor adaptation for a point to point task after stroke [13]. For the lower limbs, Emken, Benitez, and Reinkensmeyer [43] describe a novel assist-as-needed training strategy for gait rehabilitation during walking. The strategy assumes that learning a novel gait pattern can be modeled based on motor learning strategy that optimizes performance error and robotic assistance to provide the most natural assistive training. For the upper limb, Matsouka, Brewer, and Klatzky [44] provide compelling experimental data demonstrating the usefulness of a novel visual distortion technique that uses error magnification to improve motor performance of a pinching task (index finger and thumb movements). Their results provide a new method to deal with compensatory movements and learn non-use that often plagues patients after stroke. These two papers support that use of error feedback and error distortion to enhance motor learning and improving walking and pinching patterns.

Finally, Wisneski and Johnson [45] suggest that there is a need for new modeling approaches to upper limb robot-assisted therapies that support more ADL-related training. Specifically, they examine how best to implement trajectory planning for an Activity of Daily Living (ADL)-oriented approach to robot-assisted therapy with the goal of improving the ability of the ADL Exercise Robot (ADLER) to assist in the training and recovery of functional tasks such as drinking. They compare the classical minimum jerk model [46] for point-to-point movements with actual movements to perform a drinking task and speculate on what is needed for a more functional model. Their results suggest that new modeling strategies are needed in order to support more functional movements.

## Conclusion

The special issue presented nine articles that seek to capitalize on new developments in neuro-rehabilitation after stroke to improve the effectiveness of robot-assisted stroke rehabilitation. Improvements may be achieved by providing robot training environments that incorporate into their design and control strategies important elements key to inducing motor learning and cerebral plasticity such as mass-practice, feedback, task-engagement, and complex problem solving. Novel design and control strategies covered in this issue provide new methods for training more natural movements, for inducing faster motor learning control of more complex movements salient to everyday activities, and for encouraging engagement and compliance in under-supervised environments such as the home and over-burdened clinics.

## Competing interests

The author(s) declare that they have no competing interests.

## Authors' contributions

MJJ was the primary composer of the manuscript and was responsible for the intellectual content of the manuscript and gave final approval of the version to be published.

## References

[B1] (2005). Heart Disease and Stroke Statistics – 2005 Update.

[B2] Prange GB, Jannink MJA, Groothuis-Oudshoorn CGM, Hermens HJ, Ijzerman MJ (2006). Systematic review of the effect of robot-aided therapy on recovery of the hemiparetic arm after stroke. J Rehabil Res Dev.

[B3] Volpe BT, Ferraro M, Lynch D, Christos P, Krol J, Trudell C, Krebs HI, Hogan N (2002). Robotics and other devices in the treatment of patients recovering from stroke. Current Neurology & Neuroscience Reports.

[B4] Lum P, Reinkensmeyer D, Mahoney R, Rymer WZ, Burgar C (2002). Robotic Devices for movement therapy after stroke: Current status and challenges to clinical acceptance. Top Stroke Rehabil.

[B5] Fasoli SE, Krebs HI, Stein J, Frontera WR, Hogan N (2003). Effects of robotic therapy on motor impairment and recovery in chronic stroke. Archives of Physical Medicine & Rehabilitation.

[B6] Loureiro R, Amirabdollahian F, Topping M, Driessen B, Harwin W (2003). Upper limb robot mediated stroke therapy-GENTLE/s approach. Autonomous Robots.

[B7] Burgar CG, Lum PS, Shor PC, Van der Loos HFM (2000). Development of robots for rehabilitation therapy: the Palo Alto VA/Stanford experience. J Rehabil Res Dev.

[B8] Uhlenbrock D, Sarkodie-Gyan T, Reiter F, Konrad M (1997). Development of a servo-controlled Gait Trainer for the rehabilitation of non-ambulatory patients. Biomed Technik.

[B9] Hesse S, Uhlenbrock D, Werner C, Bardeleben AA (2000). Mechanized Gait Trainer for restoring gait in nonambulatory subjects. Arch Phys Med Rehabil.

[B10] http://www.hocoma.ch/index.php?lang=en&page=/pages/lokomat/lokomat_system_en.html.

[B11] Hidler J, Nichols D, Pelliccio M, Brady K (2005). Advances in the understanding and treatment of stroke impairment using robotic devices. Top Stroke Rehabil.

[B12] Johnson MJ, Wisneski KJ, Anderson J, Nathan D, Smith R (2006). Development of ADLER: The Activities of Daily Living Exercise Robot. IEEE-EMBS Biomedical Robotics (BioRob 2006).

[B13] Wei Y, Bajaj P, Scheidt R, Patton JL (2005). Visual Error Augmentation for Enhancing Motor Learning and Rehabilitative Relearning. IEEE International Conference on Rehabilitation Robotics.

[B14] Sukal TM, Dewald JPA, Ellis MD (2005). Use of a Novel Robotic System for Quantification of Upper Limb Work Area Following Stroke. IEEE International Conference on Rehabilitation Robotics.

[B15] Hornby TG, Campbell DD, Zemon DH, Kahn JH (2006). Metabolic costs and muscle activity patterns during robotic- and therapist-assisted treadmill walking in individuals with incomplete spinal cord injury. Phys Ther.

[B16] Trombly C, Trombly C (1995). Occupational Therapy of Physical Dysfunction.

[B17] Will B, Galani R, Kelche C, Rosenzweig MR (2004). Recovery from brain injury in animals: relative efficacy of environmental enrichment, physical exercise or formal training (1999–2002). Progress in Neurobioloby.

[B18] Nudo RJ (2003). Functional and structural plasticity in motor cortex: implications for stroke recovery. Physical Medicine & Rehabilitation Clinics of North America.

[B19] Bach-y-Rita P (2003). Late post-acute neurologic rehabilitation: neuroscience, engineering and clinical programs. Arch Phys Med Rehab.

[B20] Wu C, Trombly CA, Lin K, Ticke-Degnen L (1998). Effects of object affordances on reaching performance in persons with and without cerebrovascular accident. Am J Occup Ther.

[B21] Fisher BE, Sullivan KJ (2001). Activity-Dependent factors affecting poststroke functional outcomes. Top Stroke Rehabil.

[B22] Bayona NA, Bitensky J, Salter K, Teasell R (2005). The role of task-specific training in rehabilitation therapies. Topics in Stroke Rehabilitation.

[B23] Bayona NA, Bitensky J, Salter K, Teasell R (2005). Plasticity and reorganization of the uninjured brain. Topics in Stroke Rehabilitation.

[B24] You SH, Jang SH, Kim YH, Hallett M, Ahn SH, Kwon YH, Kim JH, Lee MY (2005). Virtual reality-induced cortical reorganization and associated locomotor recovery in chronic stroke: an experimenter-blind randomized study. Stroke.

[B25] Johnson MJ, Van der Loos HFM, Burgar CG, Shor P, Leifer LJ (2005). Experimental results using force-feedback cueing in robot-assisted stroke therapy. IEEE Trans on Neural Systems and Rehabilitation Engineering.

[B26] Bach y Rita P, Wood S, Leder R, Paredes O, Bahr D, Bach-y-Rita EW, Murillo N (2002). Computer assisted motivating rehabilitation for institutional, home, and educational late stroke programs. Top Stroke Rehabil.

[B27] Wood SR, Murillo N, Bach-y-Rita P, Leder RS, Marks JT, Page SJ (2003). Motivating, game-based stroke rehabilitation: a brief report. Topics of Stroke Rehabilitation.

[B28] Karni A, Meyer G, Jezzard P, Adams MM, Turner R, Ungerleider LG (1995). Functional MRI evidence for adult motor cortex plasticity during motor skill learning. Nature.

[B29] Liepert J, Bauder H, Miltner WHR, Taub E, Weiller C (2002). Treatment-induced cortical reorganization after stroke in humans. Stroke.

[B30] Classen J, Liepert J, Wise S, Hallett M, Cohen LG (1998). Rapid plasticity of human cortical movement representation induced by practice. Journal of Neurophysiology.

[B31] Calautti C, Baron J (2003). Functional neuroimaging studies of motor recovery after stroke in adults: A review. Stroke.

[B32] Schaechter JD (2004). Motor rehabilitation and brain plasticity after hemiparetic stroke. Progress in Neurobiology.

[B33] Kleim JA, Hogg TM, VandenBerg PM, Cooper NR, Bruneau R, Remple M (2004). Cortical synaptogenesis and motor map reorganization occur during late, but not early, phase of motor skill learning. J Neuroscience.

[B34] Lünenburger L, Colombo G, Riener R (2006). Biofeedback for Robotic Gait Rehabilitation. Journal of NeuroEngineering and Rehabilitation.

[B35] Schmidt H, Hesse S, KrÄuger J (2006). Gait Rehabilitation Machines based on Programmable Foot-plates. Journal of NeuroEngineering and Rehabilitation.

[B36] Reinkensmeyer DJ, Pang CT, Nessler JA, Painter CC (2002). Web-based telerehabilitation for the upper extremity after stroke. IEEE Trans Neural Systems Rehabilitation Engineering.

[B37] Johnson MJ, Van der Loos HFM, Burgar CG, Shor P, Leifer L (2003). Driver's SEAT, A car steering upper limb therapy device. Robotica.

[B38] Johnson MJ, Feng X, Johnson LM, Winters JM (2006). Potential of a Suite of Robot/Computer-Assisted Motivating Systems for Personalized, Home-Based, Stroke Rehabilitation. Journal of NeuroEngineering and Rehabilitation.

[B39] Colombo R, Pisano F, Mazzone A, Delconte C, Micera S, Chiara Carrozza M, Dario P, Minuco1 G (2006). Design Strategies to Improve Patient Motivation During Robot-Aided Rehabilitation. Journal of NeuroEngineering and Rehabilitation.

[B40] McAuley E, Duncan T, Tammen V (1987). Psychometric properties of the intrinsic motivation inventory in a competitive sport setting: a confirmatory factor analysis. Research Quartely for Exercise and Sport.

[B41] Mataric' MJ, Eriksson J, Feil-Seifer D, Winstein C (2006). Socially Assistive Robotics for Post-Stroke Rehabilitation. Journal of NeuroEngineering and Rehabilitation.

[B42] Amirabdollahian F, Loureiro RC, Gradwell E, Collin C, Harwin W, Johnson G (2006). Multivariate Analysis of the Fugl-Meyer Outcome Measures Assessing the Effectiveness of GENTLE/S Robot-Mediated Stroke Therapy. Journal of NeuroEngineering and Rehabilitation.

[B43] Emken JL, Benitez R, Reinkensmeyer DJ (2006). Human-Robot Cooperative Movement Training: Learning a Novel Sensory Motor Transformation during Walking with Robotic Assistance-as-Needed. Journal of NeuroEngineering and Rehabilitation.

[B44] Matsuoka Y, Brewer BR, Klatzky RL (2006). Using Visual Feedback Distortion to Alter Coordinated Pinching Patterns for Robotic Rehabilitation. Journal of NeuroEngineering and Rehabilitation.

[B45] Wisneski KJ, Johnson MJ (2006). Quantifying Kinematics of Purposeful Movements to Real, Imagined, or Absent Functional Objects: Implications for Modelling Trajectories for Robot-Mediated ADL Tasks. Journal of NeuroEngineering and Rehabilitation.

[B46] Flash T, Hogan N (1985). The coordination of arm movements: An experimentally confirmed mathematical model. The Journal of Neuroscience.

